# Interstitial brachytherapy combined with PARP inhibitors in the treatment of chemoresistant recurrent epithelial ovarian cancer: A case report

**DOI:** 10.3389/fonc.2022.1071383

**Published:** 2022-12-15

**Authors:** Yuan Bian, Ping Guan, Dan Li, Longjing Tan, Haowen Pang, Qinglian Wen, Ping Chen, Zhenhua Zhang

**Affiliations:** Department of Oncology, Affiliated Hospital of Southwest Medical University, Luzhou, China

**Keywords:** interstitial brachytherapy, PARP inhibitor, chemoresistance, epithelial ovarian cancer, case

## Abstract

**Background:**

Chemoresistance generally develops in patients with advanced epithelial ovarian cancer, and the prognosis is still very poor, with an expected survival time of less than one year. For this population of individuals, there is currently no standard protocol for clinical benefit.

**Case presentation:**

We report a case of an elderly woman diagnosed with stage IIIC high-grade serous ovarian cancer (HGSOC). During a follow-up time of 6 years, the patient initially received multiple sequential courses of chemotherapy with platinum-based regimens and with no maintenance therapy. Similar to most patients with advanced HGSOC, she developed platinum resistance and experienced poor treatment results with a short progression-free survival (PFS). Ultimately, we gave the patient traditional non-platinum-based chemotherapy with bevacizumab and high-dose-rate interstitial brachytherapy followed by olaparib as a maintenance therapy. Up to now, the patient did response well to the treatment, and the PFS had exceeded 12 months.

**Conclusion:**

High-dose-rate interstitial brachytherapy combination with PARP inhibitors may be an option for isolated chemoresistant recurrent epithelial ovarian cancer.

## Introduction

Most patients with epithelial ovarian cancer (EOC) are given a diagnosis of advanced-stage illness, making it rank as the fifth most typical reason for cancer-related mortality among women in the United States (1, 2). More than 70% individuals with stage III or IV disease will ultimately experience relapse, despite the fact that most of them benefit from the first-line treatments including initial debulking surgery, as well as platinum-based chemotherapy (3, 4). Platinum resistant or platinum sensitive disease are commonly used to classify patients with recurrent ovarian cancer, according to the platinum-free period of less than or more than 6 months (5). Poor prognosis and limited available treatments are associated with platinum-resistant or platinum-refractory ones, and sequential non-platinum chemotherapy is the recommended course of action in this disease setting. For these patients, however, the overall objective response rate (ORR) is modest (≤20%), the PFS is limited (median 3-4 months), and the life expectancy is short (<12 months) (6, 7). In recent years, targeted inhibitors of poly ADP ribose polymerase (PARP) have proven beneficial for multiple tumors with damaged DNA repair pathways (8–10). Apart from breast and ovarian cancer, germline or somatic aberrations in the DNA damage repair genes are found in 19% of primary prostate cancer and almost 23% of metastatic castration-resistant prostate cancer (mCRPC) and compromise genomic integrity. As such, several PARP inhibitors (PARPi) have been investigated in mCRPC patients and are effective in germline BRCA2 mutants (11). In ovarian cancer, initial researches on PARPi focused on patients who had sustained complete or partial responses to treatment with platinum (12, 13). When treating unselected platinum-resistant ones, PARPi monotherapy is only marginally effective (14–16), with response rates range from 11% in cases where homologous recombination deficit (HRD) status is unknown or negative to 20% in those with HRD (16). Therefore, we must develop a feasible collaborative strategy to improve the efficacy. Recently, technologies of proteomics, such as mass spectrometry and protein array analysis, have advanced the dissection of the underlying molecular signaling events. Within this context, proteomics analysis of ovarian cancer, as well as their adaptive responses to therapy, can uncover new therapeutic choices, which can reduce the emergence of drug resistance and potentially improve patient outcomes (17).

Here, we report a successful case of platinum-resistant isolated recurrent epithelial ovarian cancer that was treated with high-dose-rate interstitial brachytherapy (BT) and maintenance treatment using the PARP inhibitor olaparib following conventional non-platinum-based chemotherapy with bevacizumab.

## Case presentation

**Figure 1:**
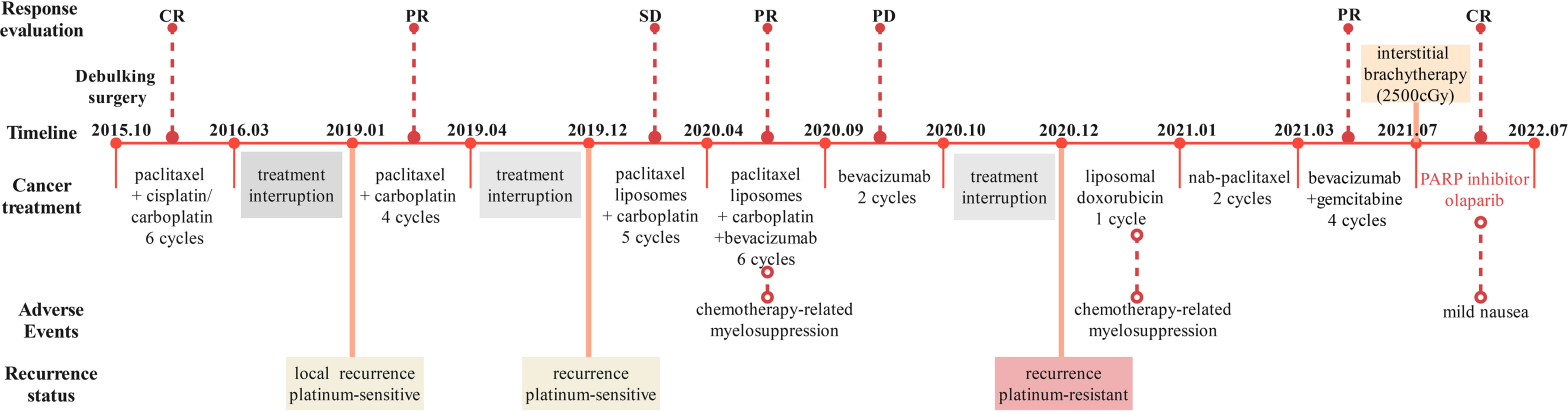
Complete timeline for treatment regimens, disease status and patient treatment response.

The patient, a 63-year-old woman from Sichuan, China, arrived at our hospital in October 2015, who complained of anal swelling, with no special past medical history. Then she had primary debulking surgery after being diagnosed with ovarian cancer. Rectal invasion was identified during the initial surgery, which included an abdominal hysterectomy, bilateral adnexectomy, and omentectomy, thus the upper rectal focus was then resected. Histopathological diagnosis was high-grade serous carcinoma (HGSC) and the patient was staged as having stage IIIC ovarian cancer according to the International Federation of Gynecology and Obstetrics (FIGO) criteria. According to the NCCN guideline, we gave the patient post-operative chemotherapy, and the timeline of the treatment process is shown in **Figure 1**. No tumor was detected by CT after 6 cycles of TP/TC therapy (175 mg/m^2^ paclitaxel plus 75/mg^2^ cisplatin, or 175 mg/m^2^ paclitaxel plus carboplatin area under the curve (AUC) 5), so she was considered to have achieved complete clinical response (CR). Regrettably, the patient discontinued treatment and was not followed up regularly.

**Figure 2:**
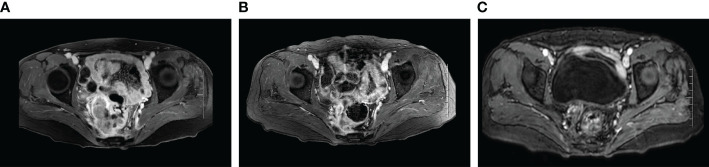
MRI images of local recurrent lesions during treatment. **(A)** Local recurrence of the last progression (January 2019). **(B)** MRI images after the last 6 cycles of non-platinum-based chemotherapy combined with bevacizumab (July 2021). **(C)** MRI images of 12 months after maintenance treatment using the PARP inhibitor Olaparib (July 2022).

The patient was readmitted in January 2019 with vaginal bleeding and lower abdominal pain. Magnetic resonance imaging (MRI) and [^18^F] fluorodeoxyglucose (FDG) –positron emission tomography (PET) showed that there was a mass on the right side of the vaginal stump and the right posterior rectal wall, accompanied by increased glucose metabolism (**Figure 2A**). Local pelvic recurrence of ovarian cancer was considered. Platinum-based chemotherapy (paclitaxel combined with carboplatin) is again selected according to platinum-sensitive relapse when satisfactory tumor reduction surgery could not be performed. After four courses of TC, a partial response (PR) was observed and chemotherapy was discontinued because it was poorly tolerated. After a 9-month platinum-free interval, the patient suffered from vaginal bleeding again, this time along with enlarged vaginal stump lesions. Five cycles of paclitaxel liposomes in conjunction with carboplatin were administered, with a result graded as stable disease (SD). Afterward, the efficacy was considered to be PR when we added bevacizumab to the original treatment for the other 6 cycles.

**Figure 3:**
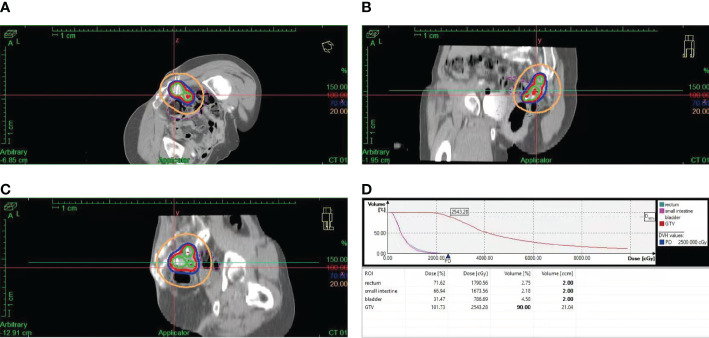
Three-dimensional conformal dose assessment for interstitial BT. **(A)**. Cross-sectional image. **(B)**. Sagittal image. **(C)**. Coronal image. **(D)**. Dose-volume histogram (DVH).

**Figure 4:**
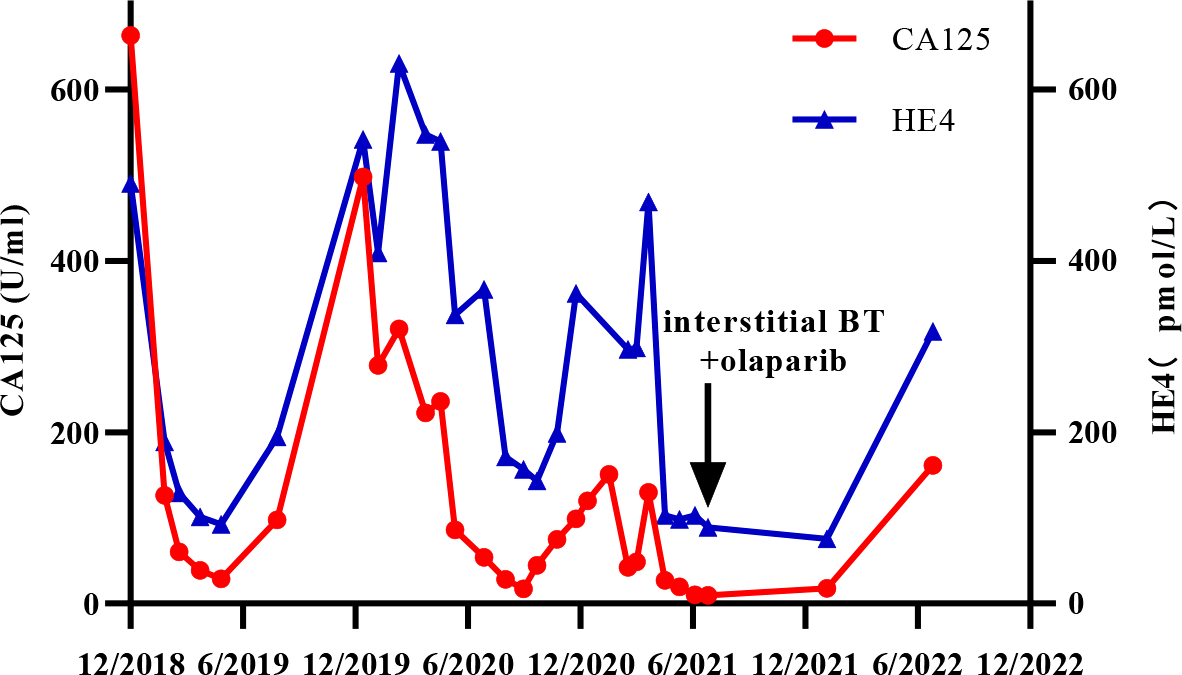
Changes in serum CA125 and HE4 levels during the whole process of treatment.

Unfortunately, the patient’s condition deteriorated again after two months of platinum-free treatment, including maintenance therapy of bevacizumab. Considering platinum-resistant relapse, doxorubicin liposomal was given in a clinical trial in December 2020, but halted after only one cycle due to the side-effects such as Grade III myelosuppression and intestinal obstruction. Given this situation, we administered the patient albumin-paclitaxel-based palliative chemotherapy (125mg/m^2^ d1.8.15 q28d, lasted 2 cycles) in January 2021. Then, the treatment scheme was altered to the combination of bevacizumab and gemcitabine due to the inadequate retraction of the focus on imaging. After 4 cycles of therapy, the focus was significantly reduced and reached PR. Considering the recurrence of the same isolated pelvic lesion, the radiation oncologist of our hospital performed interstitial brachytherapy (BT) of right sacrococcygeal residual lesions in July 2021 with a prescription dose was 2500cGy. **Figure 3** shows the three-dimensional conformal dose assessment for interstitial brachytherapy. Following the completion of radiotherapy, the patient received individual therapy with PARP inhibitor (olaparib) for maintenance due to homologous recombination deficiency (HRD). Currently, the patient remained in CR at the last imaging follow-up in July 2022, even though tumor markers (serum CA125 and HE4) had started to slowly increase (**Figure 4**). At the same time, the patient did not experience any substantial radiation damage, such as radiation proctitis or radiation cystitis. In **Figure 2**, we display the radiographic changes occurred in our patient during treatment.

## Discussion

Ovarian cancer, the most lethal gynecologic malignancy, has a variety of genotypes and phenotypes within each histological subtype, which of all might affect the biological process and chemotherapeutic response (18, 19). Additionally, the illness process is dynamic, with alterations in genetics and epigenetics that have evolved throughout time and in various metastatic disease locations (20, 21). The patient with HRD in this case has repeated multi-line chemotherapy (>3) but no maintenance therapy. Just as most patients with advanced HGSOC, she developed platinum resistance and experienced poor treatment results with a short progression-free survival (PFS). Fortunately, each recurrence in our patient was at the same site and was an isolated lesion.

To date, there is no adequate curative regimen for platinum-resistant ovarian cancer, thus patients now must endure sequential non-platinum chemotherapy or enroll in clinical trials. Overall, the prognosis for these patients is still generally poor.

We know that ovarian cancer has been shown to be sensitive to radiotherapy (22) that was once employed as an adjuvant treatment after initial surgery (23), and whole abdominal radiation had been considered a good option for treating small residual lesions (24, 25). However, due to its toxicity and the development of platinum-based chemotherapeutic agents, whole abdominal radiation is soon no longer routinely used in the clinical management of ovarian cancer and only used for the treatment of isolated lesions (26, 27). Interstitial brachytherapy, a form of radiotherapy, has achieved excellent success treating a variety of solid cancers (28, 29). Because the recurrent lesion is solitary in our patient, interstitial brachytherapy can provide a greater dosage to control the tumor and achieve good therapeutic benefits of CR without having the negative side effects of traditional radiotherapy, which is essential for improving the patient’s PFS.

Recent studies have shown that PARP inhibitors are highly effective for the maintenance treatment of platinum-sensitive recurrent ovarian cancer and greatly reduce the risk of disease progression and patient mortality (12, 30, 31). For recurrent platinum-resistant illness associated with HRD or pathogenic mutations in BRCA1 and BRCA2 (BRCA1/2), PARP inhibitors such as niraparib, olaparib, and rucaparib are also beneficial in various degrees (16, 32, 33). Remarkably, when mutations occur within DNA repair pathways, there is an increased risk of chemotherapy resistance. Among evaluated PARP inhibitors, olaparib, niraparib, and rucaparib are approximately 100-fold more potent than veliparib, while talazoparib has the most enhanced trapping potency. It has been suggested a correlation between increased PARP trapping and high myelosuppression, which results on variation in dosing among PARP inhibitors (34). In our patient, olaparib was used as maintenance therapy due to HRD after interstitial brachytherapy, and we achieved a promising PFS of more than 12 months. This result is significantly greater than several previous results(median PFS of 2.9 months) with single-agent olaparib therapy in platinum-resistant ovarian cancer (PROC), and close to that in patient with HRD when the response to platinuam therapy is not taken into consideration (median PFS of 13.6 months) (16, 32, 33).

In addition, combining PARPi with radiotherapy to treat tumors may be a promising strategy. Acting as a radiosensitizer to inhibit the proliferation of tumor cells, PARPi can enhance the anti-tumor immune response by boosting CD8 ^+^ T lymphocytes and activating the STING/TBK1/IRF3 pathway (35), and the combined treatment demonstrated superior anti-tumor effects when compared to the PARPi or radiotherapy (35–37). There is evidence that BRCA deficiency may induce a STING-dependent innate immune response, by inducing type I interferon and pro-inflammatory cytokine production. Beyond this, clinical models have also demonstrated that PARP inhibition inactivate GSK3 and upregulate PD-L1 in a dose-dependent manner. Consequently, T-cell activation is being suppressed, resulting in enhanced cancer cell apoptosis (38). On the other hand, immunotherapy, a popular topic in treatment of tumors, has been shown to have a mutually reinforcing effect in terms of efficacy in combination with chemoradiotherapy (39). Increasing PD-L1 expression, inducing immunogenic cell death (ICD), and inhibiting tumor development, as well as extending the life period may all be achieved, by activating effector T cells and modifying the tumor microenvironment, through combining immunotherapy with PARPi and radiation treatment (40). Although there is presently insufficient data to evaluate if these therapies can also help treat PROC, the aforementioned findings gave us new insight into the therapeutic strategy in the future.

To our knowledge, this is the first case of interstitial brachytherapy combined with PARP inhibitors being used to treat PROC. The patient received platinum-based chemotherapy after surgery and eventually developed a platinum-resistant relapse during the next few years, and we tried to use traditional non-platinum-based chemotherapy combined with bevacizumab and interstitial brachytherapy, followed by maintenance treatment with the PARP inhibitor olaparib. To date, the patient has thus far maintained good health, with no disease progression over the past 12 months and effective management of tumor markers CA125 and HE4 (tumor markers started to slowly increase at the last follow-up in June 2022).

## Conclusion

Due to the poor prognosis, short progression-free survival, and lack of established treatment regimens, PROC is a challenging problem for oncologists. Interstitial brachytherapy is commonly used to some solid cancers, while PARP inhibitors are used as a maintenance therapy for platinum-sensitive recurrent ovarian cancer, but it is uncertain if PROC will also benefit from them. Recently, a clinical trial on the effectiveness of re-introduction or continuation of PARP inhibitors after local therapy for oligo-metastatic progression in patients with relapsed ovarian cancer was presented at the European Society of Oncology. After local therapy, patients with oligo-metastatic progression were given PARP inhibitors, which led to a provocative result, with a median PFS of 11.5 months (41). In our case, the patient, despite being platinum-resistant, achieved a similar result after receiving high-dose-rate interstitial brachytherapy and olaparib as a maintenance therapy, which is quite encouraging. We anticipate further validation of the effectiveness of this treatment in more clinical settings in the future.

## Data availability statement

The original contributions presented in the study are included in the article/Supplementary Material. Further inquiries can be directed to the corresponding authors.

## Ethics statement

Written informed consent was obtained from the individual (s) for the publication of any potentially identifiable images or data included in this article.

## Author contributions

All authors listed have made a substantial, direct, and intellectual contribution to the work and approved it for publication.
